# Activation of cell-penetrating peptide fragments by disulfide formation

**DOI:** 10.1007/s00726-020-02880-x

**Published:** 2020-07-31

**Authors:** Raheleh Tooyserkani, Wojciech Lipiński, Bob Willemsen, Dennis W. P. M. Löwik

**Affiliations:** Radboud University Nijmegen, Institute for Molecules and Materials, Bio-Organic Chemistry, Heyendaalseweg 135, 6525 AJ Nijmegen, The Netherlands

**Keywords:** Cell-penetrating peptide, Cellular uptake, Disulfide conjugation, Tat, Pep-3, Penetratin

## Abstract

Three cell-penetrating peptides (CPPs), Tat, Pep-3 and penetratin, were split into two parts and each fragment was terminated with a cysteine residue, to allow disulfide bridge formation, as well as a fluorescent label, for visualization and quantitative analysis. After disulfide formation between two complementary CPP fragments, cellular uptake of the resulting conjugates was observed. As confirmed by in vitro experiments, the conjugated peptides showed uptake activity comparable to the native CPP sequences, while the truncated peptides were hardly active. Until now, this split CPP strategy has only been demonstrated for oligo-arginine CPPs, but here we demonstrate that it is also applicable to other cell-penetrating peptides. This wider applicability may help in the design of new activatable cell-penetrating peptides for, e.g., targeted drug delivery.

**Electronic supplementary material:**

The online version of this article (10.1007/s00726-020-02880-x) contains supplementary material, which is available to authorized users.

## Introduction

**Fig. 1:**
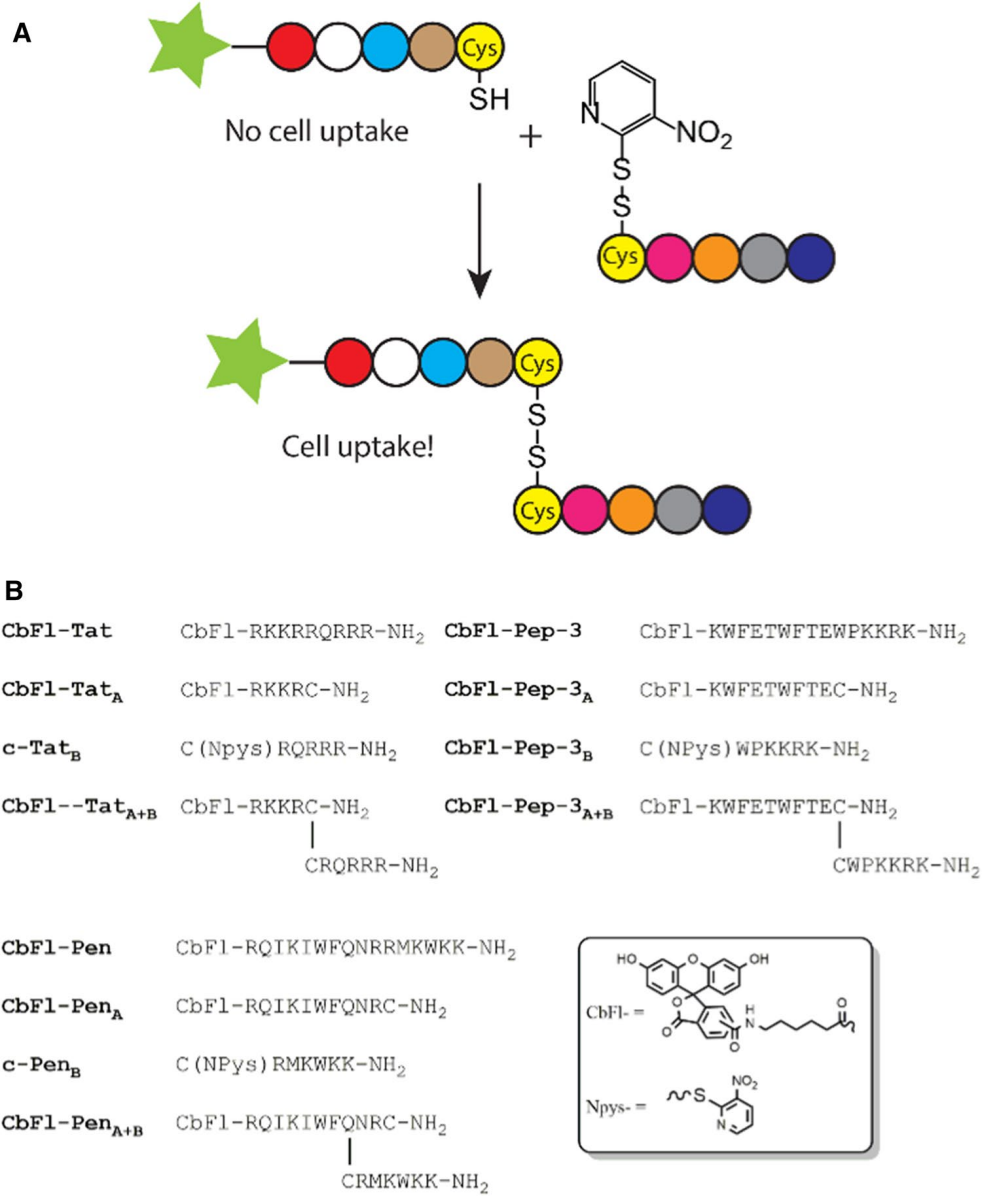
General concept of the activation of split CPPs (**a**) and the Tat, Pep-3 and Pen-based peptide sequences used in this study (**b**)

In the search for new, efficient delivery methods of therapeutic molecules and particles into cells, cell-penetrating peptides (CPPs) emerged 30 years ago as extraordinary vector molecules (Copolovici et al. 2014). They can facilitate transmembrane transport through a receptor-independent pathway and without inducing toxicity. However, despite a great number of both natural and synthetic CPPs that have been discovered, varying in their chemical structure and exhibiting different properties, the common use of CPPs as delivery vectors is hampered due to a number of practical difficulties (Feni and Neundorf 2017; Shi et al. 2014). Firstly, because the mechanism of cellular uptake remains elusive in many cases, and as it seems to be significantly dependent on the type of cargo, type of cells and experimental conditions, it is very difficult to develop an all-embracing delivery solution based on CPPs (Madani et al. 2011). Secondly, and probably even more important, CPPs are taken up non-selectively in a great variety of cells. Hence, several strategies have been developed to improve the selectivity of CPP-based delivery platforms. For example, CPPs or CPP-based delivery platforms can be coupled to targeting ligands (e.g., hyaluronic acid, (Yamada et al. 2015) folic acid (Li et al. 2016), transferrin (Sharma et al. 2016), bombesin (Nallely et al. 2013), RGD or NGR sequence (Xie et al. 2016)) or can be incorporated into macromolecular carriers (e.g., nanoparticles (Morshed et al. 2016), polymers (Chen et al. 2012) or liposomes (Yang et al. 2014)). In addition, a very promising strategy is the reversible inactivation of CPPs and creating so-called activatable cell-penetrating peptides (aCPPs), of which membrane-penetrating abilities can be elicited by an external trigger [e.g., enzymatic cleavage (Bode et al. 2015; Jiang et al. 2004), pH change (Jin et al. 2013) or irradiation (Hansen et al. 2012; Wang et al. 2018)] at the site of action. In our own laboratory, we have developed a strategy that deploys the conjugation of inactive fragments (Fig. 1a). We showed that four-mer and five-mer fragments of the well-known CPPs octa- and nona-arginine show very little uptake in comparison to the full length peptide, but that these truncated peptides regained their cellular uptake ability upon reconstitution to a molecule containing at least eight arginine residues. This reconstitution was achieved both covalently, by disulfide bond formation (Bode et al. 2014) and inverse electron demand Diels–Alder bio-conjugations (Bode et al. 2019), as well as non-covalently through the interaction of a leucine zipper pair. (Bode et al. 2017).

To date, this split CPP strategy has only been demonstrated for oligo-arginines and these are not always the best option when choosing a CPP. Therefore, we wanted to investigate whether our strategy to construct activatable split CPPs is also applicable to fundamentally different CPPs than oligo-arginines. To this purpose, we selected three CPPs from different classes: Tat, a cationic CPP still closely related to octa- and nona-arginine, but also Pep-3 and penetratin (Pen), both amphipathic CPPs and quite distinct from Tat and the related oligo-arginines. We chose disulfide formation as the method of conjugation for the fragments because of its synthetic simplicity. Tat peptide is a very well-studied, cationic CPP derived from the HIV TAT protein (Brooks et al. 2005). Pep-3 is a rationally designed primary amphipathic CPP comprising a tryptophan/phenylalanine-rich hydrophobic domain and a lysine/arginine-rich hydrophilic motif (Morris et al. 2007). Penetratin is derived from the third α-helix of the homeodomain of Antennapedia, a Drosophila homeoprotein (Derossi et al. 1994).

## Results and discussion

As mentioned above, the aim of this study was to investigate the compatibility of our split CPP inactivation–activation strategy with three different CPPs (Fig. 1b). To this end, we synthesized Tat (RKKRRQRRR), Pep-3 (KWFETWFTEWPKKRK) and penetratin, (RQILIWFQNRRMKWKK). Moreover, we labeled all three N-terminally with 5(6)-carboxyfluorescein (Cbfl) in combination with an ε-aminohexanoic acid (6Ahx) spacer to yield CbFl-Tat, CbFl-Pep-3 and CbFl-Pen, respectively, that serve as reference CPPs. We chose to divide the Tat sequence into two moieties such that each fragment contained four basic amino acids following our earlier strategy that we used for octa-arginine (Bode et al. 2014). The Pep-3 sequence we split to yield a hydrophobic and a hydrophilic fragment, comprising, respectively, nine and six amino acids of the full length sequence. We decided to fragment Pen in a 10-mer N-terminal and a 6-mer C-terminal fragment, which were shown by Fisher et al*.*, in a structure activity relationship study, to give minimal uptake compared to the full length Pen (Fischer et al. 2000). The N-terminal fragments, also all labeled with Cbfl and connected via a 6Ahx spacer, had a cysteine residue added to their C-terminus to give CbFl-Tat_A_, CbFl-Pep-3_A_ and CbFl-Pen_A_. The C-terminal parts, c-Tat_B_, c-Pep-3_B_ and c-Pen_B_, had a cysteine residue added to their N-terminus with a 3-nitro-2-pyridinesulfenyl (Npys) group on the thiol group that allowed for a facile preparation of the corresponding reconstituted peptides, CbFl-Tat_A+B_, CbFl-Pep-3_A+B_ and CbFl-Pen_A+B_. Mixing both parts of the split CPPs at pH 7.4 resulted in a smooth formation of the disulfide-linked peptides comprising all amino acids of the native sequences. (We prepared Pen_A+B_ in DMF because the Pen_A_ fragment would not dissolve in aqueous media.) All synthesized peptides were purified by preparative HPLC, and all products were obtained with a purity greater than or equal to 95%, as assessed by analytical reversed phase HPLC. All sequences are summarized in Fig. 1b.

**Fig. 2:**
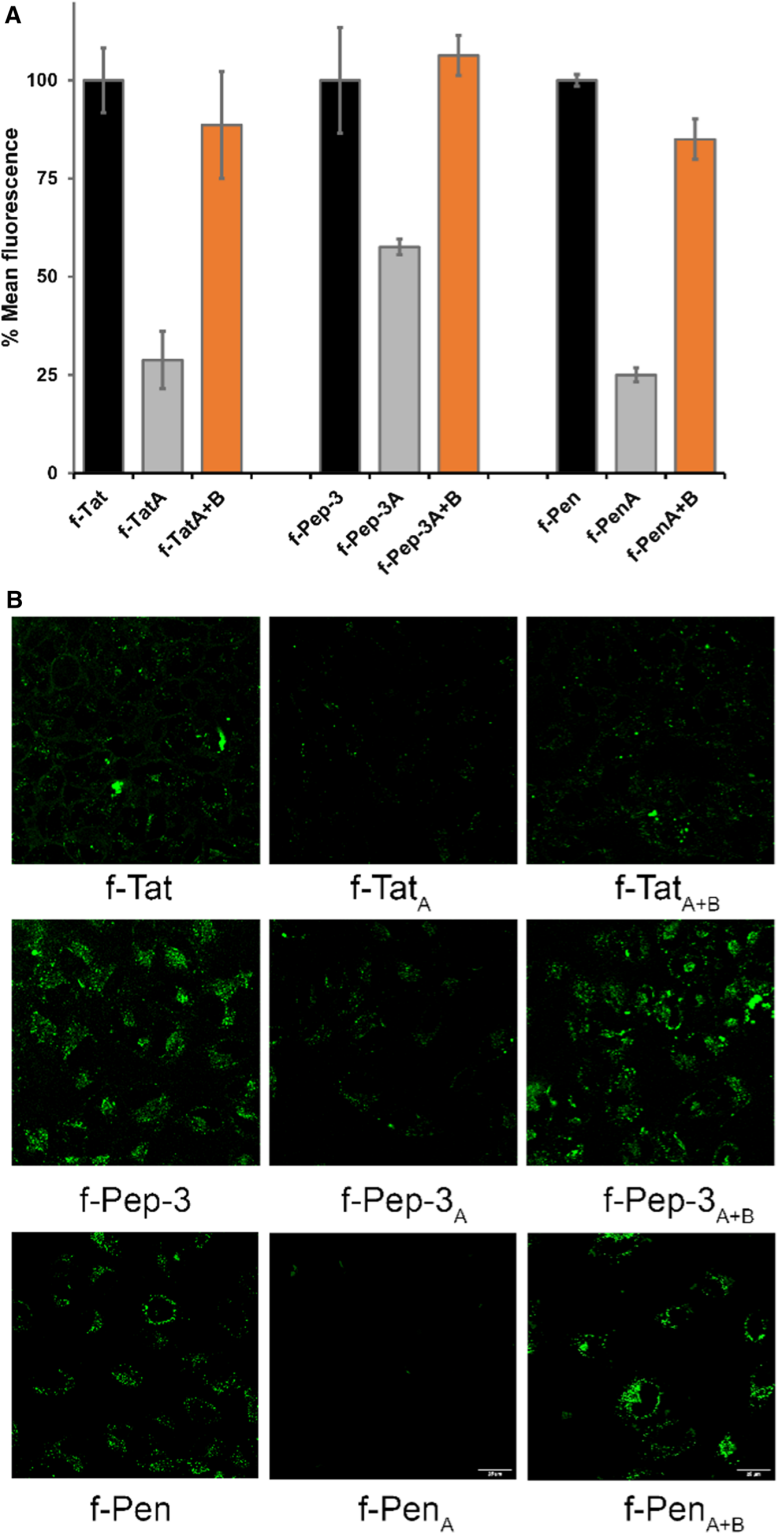
Summary of flow cytometry results **a** for the Tat, Pep-3 and penetratin-derived peptides and the corresponding confocal fluorescence images (**b**). The uptake was studied by incubating the peptides at 5 µM in serum-containing medium, for 30 min at 37 °C. Uptake of CbFl-Tat, CbFl-Pep-3 and CbFl-Pen were each set to 100%

With these peptides in hand, we first determined the uptake efficiency of the Cbfl labeled peptides at a concentration of 5 µM by incubating them with HeLa cells in serum containing medium for 30 min at 37 °C. Like in our earlier study, we quantified uptake using flow cytometry and visualized it by confocal fluorescence microscopy (Bode et al. 2014). Both flow cytometry and confocal microscopy revealed that the reconstituted peptide CbFl-Tat_A+B_ was internalized with an efficiency that is comparable to the native peptide, CbFl-Tat, as is shown in Fig. 2. Moreover, the truncated peptide CbFl-Tat_A_ expressed relatively low activity in comparison to CbFl-Tat and CbFl-Tat_A+B_, comparable to what we found for truncated oligo-arginine in our earlier study (Bode et al. 2014).

Hence, we can conclude that our activation strategy based on the ligation of fragments can be successfully applied for the Tat peptide. However, it must be noted that the Tat peptide is very closely related to oligo-arginine, which we studied before and in which the presence of charge is a dominant factor that influences CPP behavior. Therefore, we continued with the more distinct Pep-3 and penetratin, from the class of so-called primary and secondary amphipathic CPPs (Madani et al. 2011; Zaro and Shen 2015).

As for the Tat-derived peptides, the uptake efficiency of the Pep-3 derived peptides was quantified at a concentration of 5 µM in HeLa cells using flow cytometry and visualized by confocal fluorescence microscopy (Fig. 2). Flow cytometry results suggest that the N-terminal fragment CbFl-Pep-_3A_ is only slightly less (58%) efficiently taken up by the HeLa cells than the full length sequence CbFl-Pep-3 and the reconstituted peptide CbFl-Pep-3_A+B_. Possibly, we could have split the CPP into two different parts to achieve a better inactivation. Nevertheless, the reconstituted CbFl-Pep-3_A+B_ was taken up as efficiently as the native CbFl-Pep-3, which suggests that our strategy of activation by reconstitution is also viable for CPP distinct from the arginine-rich ones.

Next, we moved to penetratin of which it was fortunately already known, from a SAR study by Fisher et al., (Fischer et al. 2000) that the fragments we split the peptide into are barely taken up. Indeed, we corroborated that the truncated CbFl-Pen_A_ is hardly taken up in HeLa cells at a concentration of 5 µM (Fig. 2) Satisfyingly, the reconstituted CbFl-Pen_A+B_ was able to enter HeLa cells almost as efficiently as the native full length CbFl-Pen.

**Fig. 3:**
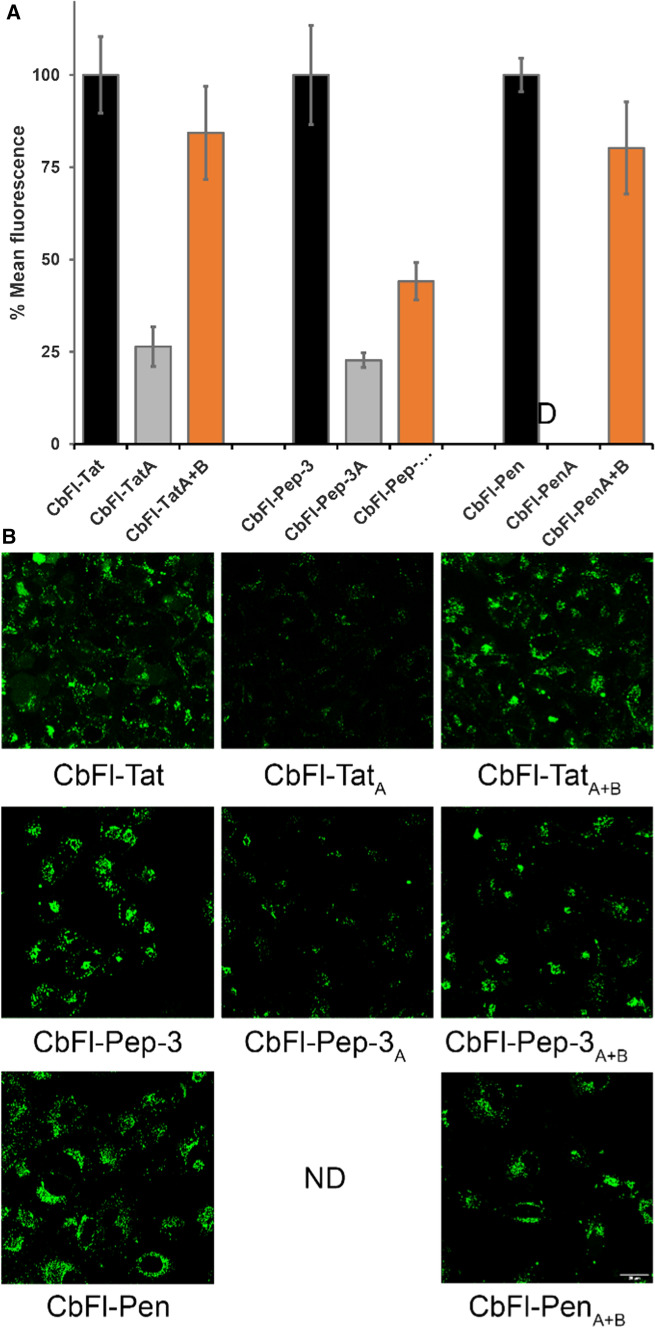
Summary of flow cytometry results **a** for the Tat, Pep-3 and penetratin-derived peptides and the corresponding confocal fluorescence images (**b**). The uptake was studied by incubating the peptides at 20 µM in serum-containing medium, for 30 min at 37 °C. Uptake of CbFl-Tat, CbFl-Pep-3 and CbFl-Pen were each set to 100%. ND: not determined because of insolubility of the peptide

Because it is known that the cellular uptake behavior of CPPs can be concentration dependent, we decided to test our peptides also at a concentration of 20 µM, as we did in our earlier study with oligo-arginines (Bode et al. 2014; Brock 2014). Again, we studied the peptides by incubating them with HeLa cells in serum containing medium for 30 min at 37 °C and we quantified uptake using flow cytometry and visualized it by confocal fluorescence microscopy as is shown in Fig. 3.

We found that also at 20 µM, reconstituted peptide CbFl-Tat_A+B_ was taken up with an efficiency comparable to the native peptide CbFl-Tat and that the truncated peptide CbFl-Tat_A_ was poorly taken up compared to the native CbFl-Tat and reconstituted CbFl-Tat_A+B_. The only difference with the incubation at 5 µM was that the activity of both CbFl-Tat and Tat_A+B_ was much higher, which was expected at this higher concentration. For Pep-3, however, at 20 µM the reconstituted peptide CbFl-Pep-3_A+B_ was taken up only half as efficiently as the native peptide, while the activity of short hydrophobic N-terminal peptide CbFl-Pep-3_A_ displayed 20% of the activity of CbFl-Pep-3. According to confocal microscopy analysis, these differences did not seem to be as pronounced, but nevertheless follow the same trend as the FACS results as can be seen in Fig. 3. This poor activation at 20 µM cannot be ascribed to toxicity, as none of the peptides in this study proved to be highly toxic (see supporting information). Finally, for penetratin we found again that the disulfide-bridged CbFl-Pen_A+B_ was taken up comparably to the native CbFl-Pen. However, at the concentration of 20 µM the N-terminal part CbFl-PenA turned out to be insoluble in aqueous media. Also, the addition of small amounts of organic co-solvent could not keep the peptide in solution at this concentration. Therefore, we could not determine the uptake of this particular fragment. Possibly, when we would attach CbFl-Pen_A_ to a much larger cargo, the lower solubility would be less of an issue.

## Conclusion

In conclusion, we have shown that the strategy of CPP activation based on reconstitution from shorter fragments via disulfide formation is also applicable to CPPs other than oligo-arginines. We postulate that all cationic CPPs, whose activity is not strictly related to their secondary structure, may be activated by connecting truncated fragments. On the other hand, experiments with Pep-3-derived peptides did not lead to an unambiguous conclusion. At 5 µM, the reconstituted peptide was taken up equally well as the native peptide. Somehow, the truncated version still displayed considerable uptake properties. Similar behavior has been observed for penetratin when truncated (Fischer et al. 2000). A better inactivation might be achieved by splitting the peptide at a different position. The penetratin derivatives showed uptake behavior similar to those of oligo-arginine and Tat. Truncated Pen_A_ is hardly taken up, while the disulfide bridged fragments are taken up as efficiently as the native full length penetratin itself. These results show that our split CPP strategy is also applicable beyond mere cationic CPPs such as oligo-arginines and Tat.

At 20 µM, the strategy was less as successful in the case of Pep-3. Apparently, a different uptake mechanism might apply in this case where more specific interactions with the cell membrane or the formation a secondary structure could be of more importance for the induction of cellular uptake. Perhaps, the influence of the additional cysteine residues and concomitant disulfide bridge might be too disruptive on the Pep-3 activity. We are also investigating other ligation methods for reconstitution to possibly resolve this problem (Bode et al. 2019). For both Tat and penetratin, our strategy of activation by reconstitution worked, although the uptake of the N-terminal penetratin fragment could not be established due to its insolubility in aqueous media at 20 µM. Concluding, we think our strategy could find applications in targeted drug delivery based on activatable cell-penetrating peptides, especially when combined with other bio-orthogonal conjugation chemistry that we have shown to work for oligo-arginines (Bode et al. 2019).

## Experimental

### General

Unless stated otherwise, all chemicals were purchased from commercial sources and all reactions were carried out under ambient atmosphere. Breipohl resin was obtained from Bachem (Bubendorf, Switzerland). Fmoc-amino acids were purchased from Novabiochem (EMD Chemicals, Gibbstown, USA.), Fmoc-6-Ahx-OH was acquired from Iris Biotech GMBH (Marktredwitz, Germany) and carboxyfluorescein was obtained from Acros Organics (Geel, Belgium). MilliQ was doubly deionized using a Labconco Water Pro PS purification system (18.1 MΩ).

Analytical HPLC was performed on a Shimadzu LC-20A system, equipped with a C18 ReproSil column (15 cm × 3 mm, 3 μm). A gradient program was used, 5–100% of phase B in phase A during 40 min (phase A: 100% H2O + 0.1% TFA, phase B: 100% MeCN + 0.1% TFA), flow 0.4 ml/min.

Preparative HPLC was performed on a Shimadzu LC-20A system, equipped with an NX-C18 column (15 cm × 21.2 mm, 10 µm). A gradient program was used 5–100% of phase B in phase A (phase A: 100% H2O + 0.1% TFA, phase B: 100% MeCN + 0.1% TFA), flow 6.0 ml/min.

Mass spectra were acquired on a Thermo Finnigan LCQ Advantage Max (ESI-Q) system or on a Bruker Microflex LRF (MALDI-ToF) system (with α-cyano-4-hydroxycinnamic acid matrix).

### Peptide synthesis

Peptides were synthesized on polystyrene Breipohl amide resin using a Labortec SP4000 peptide synthesizer employing a standard Fmoc solid-phase peptide synthesis (SPPS) protocol. In brief, the resin was swollen in DMF for 60 min prior to use. The Fmoc protecting groups were removed by shaking the resin with piperidine in DMF (20%, v/v) for three times 6 min. The desired sequence of amino acids was coupled to the resin using Fmoc-amino acid or Boc-Cys(Npys)-OH or 5(6)-carboxyfluorescein (3.0 equiv), diisopropylcarbodiimide (DIPCDI, 3.3 equiv) and *N*-hydroxy benzotriazole (HOBt, 3.6 equiv), dissolved in DMF. Peptide couplings were monitored using the Kaiser test. After the final Fmoc removal, the resin was subsequently washed with DMF (3 times), dichloromethane (3 times), methanol (3 times), dichloromethane (3 times) and diethyl ether (3 time) and air-dried for at least 2 h. The peptides were cleaved from the resin by suspension in a mixture of TFA/H2O/triisopropylsilane/thioanisole (90:5:2.5:2.5 v/v/v/v) for 2–5 h. The free peptides were precipitated in Et_2_O, and the crude products were air dried overnight. The crude peptides were purified by reversed-phase HPLC and subsequently lyophilized yielding a yellow powder (CbFl labeled peptides) or cream powder (peptides with N-terminal Cys(Npys)). Purity was evaluated by analytical reversed-phase HPLC and identity confirmed by mass spectrometry. All peptides were obtained with a purity greater than or equal to 95%, as assessed by analytical reversed-phase HPLC.

### Preparation of extended peptides

Disulfide linkage between the activating and truncated peptides was achieved by dissolving a fluorescein-labeled short peptide with a free C-terminal cysteine in 0.1 M degassed sodium phosphate buffer (pH 7.4) and subsequently mixing it with 1.1 equiv. of the corresponding short peptide with an N terminal Npys-protected Cys. During this reaction, the final peptide concentration was 1–5 mM. The reaction was monitored by reversed-phase HPLC. At the end of the reaction, the resulting peptides were purified by reversed-phase HPLC. After lyophilization, the peptides were obtained as yellow powders. All peptides were obtained with a purity greater than or equal to 95%, as assessed by analytical reversed-phase HPLC. The analytical details are summarized in table S1.

### Cell culture

HeLa cells were maintained in sterile conditions in DMEM supplemented with 10% heat-inactivated fetal bovine serum. All cells were incubated at 37 °C in a humidified atmosphere of 7.5% CO_2_. Cells were passaged every 2–4 days.

### Flow cytometry

HeLa cells were seeded in 24-well plates 1 or 2 days prior to the experiment (40,000 or 80,000 cells/well). On the day of the experiment, cells were incubated with the peptide solutions (5 μM or 20 μM in medium) for 30 min at 37 °C. After washing the cells with PBS buffer (pH 7.4), cells were detached by trypsinization for 5 min, spun down and resuspended in 200 μL FACS buffer (PBS + 0.1 wt% BSA). The fluorescence was measured using a Beckman Coulter FC500 flow cytometer. Results were based on 10,000 gated cells. Representative histograms are given in Figs. S1-3.

### Confocal microscopy

HeLa cells were seeded in chambered coverslips 1 or 2 days prior to the experiment (25,000 or 50,000 cells/well). On the day of the experiment, cells were incubated with the peptide solutions (5 μM or 20 μM in medium) for 30 min at 37 °C. Cells were washed twice after incubation with medium, and living cells were analyzed immediately by confocal microscopy using a Leica Microsystems SP8 X confocal microscope equipped with an HC PL APO 63x/1.20 W lens. Fluorescein was excited by white light laser at 488 nm and emission was collected between 500 and 550 nm.

### Cytotoxicity assay

Cytotoxic activity of the CPPs was evaluated for the Hela cells with the WST-8 reagent. Accordingly, Hela cells were seeded at a density of 10,000 cells per well into a 96-well plate and incubated overnight at 37 °C in 7.5% CO_2_ atmosphere. At a confluency of 70%, CPPs were added at final concentrations of 5 and 20 µM with three replicates at each concentration. A medium solution without peptide was taken as a background measurement. After 30 min of incubation at 37 °C in cell medium, supernatants were discarded and cells were incubated with WST-8 reagent for 4 h. Absorbance was measured at 450 nm against 650 nm as a reference wavelength. Results are shown in Fig. S4.

## Electronic supplementary material

Below is the link to the electronic supplementary material.

Additional preparation procedures and characterization (viability study, HPLC, Mass analysis) of the peptides, can be found in the electronic supplementary information (PDF 1664 kb)
