# Supplementary material for: Activation of cell-penetrating peptide fragments by disulfide formation
Source: Amino Acids. 2020 Jul 31;52(8):1161–8. doi: 10.1007/s00726-020-02880-x (PMC7497323; doi:10.1007/s00726-020-02880-x)

- SUPPORTING INFORMATION -

Activation of Split Cell Penetrating Peptides by Reconstitution

Raheleh Tooyserkani, Wojciech Lipiński, Bob Willemsen and Dennis W. P. M. Löwik*

Summary of analytical details

Table S1. Characterization of the synthesized peptides

peptide	calc. monoisotopic mass	main MS peak		RT (min)	purity
**CbFl-Tat**	1809.01	1811.95	$[M+H]^+$	14.2	99%
**CbFl-Tat$_A$**	1159.56	1161.39	$[M+H]^+$	14.8	99%
**c-Tat$_B$**	1026.48	627.9	$[M+2TFA+2H]^{2+}$	11.5	98%
**CbFl-Tat$_{A+B}$**	2031.05	2033.61	$[M+H]^+$	14.1	99%
**CbFl-Pep-3**	2567.25	857.3	$[M+3H]^{3+}$	19.4	98%
**CbFl-Pep-3$_A$**	1846.74	925.1	$[M+2H]^{2+}$	21.9	95%
**c-Pep-3$_B$**	1097.54	550.5	$[M+2H]^{2+}$	13.8	99%
**CbFl-Pep3$_{A+B}$**	2788.28	930.9	$[M+3H]^{3+}$	19.1	99%
**CbFl-Pen**	2715.44	453.9	$[M+6H]^{6+}$	17.3	99%
**CbFl-Pen$_A$**	1945.53	981.7	$[M+2H]^{2+}$	18.3	98%
**c-Pen$_B$**	1131.53	378.1	$[M+3H]^{3+}$	14.0	98%
**CbFl-Pen$_{A+B}$**	2921.46	490.8	$[M+6H]^{6+}$	17.8	99%

See page S8-S13 for chromatograms and mass spectra.

FACS analyses

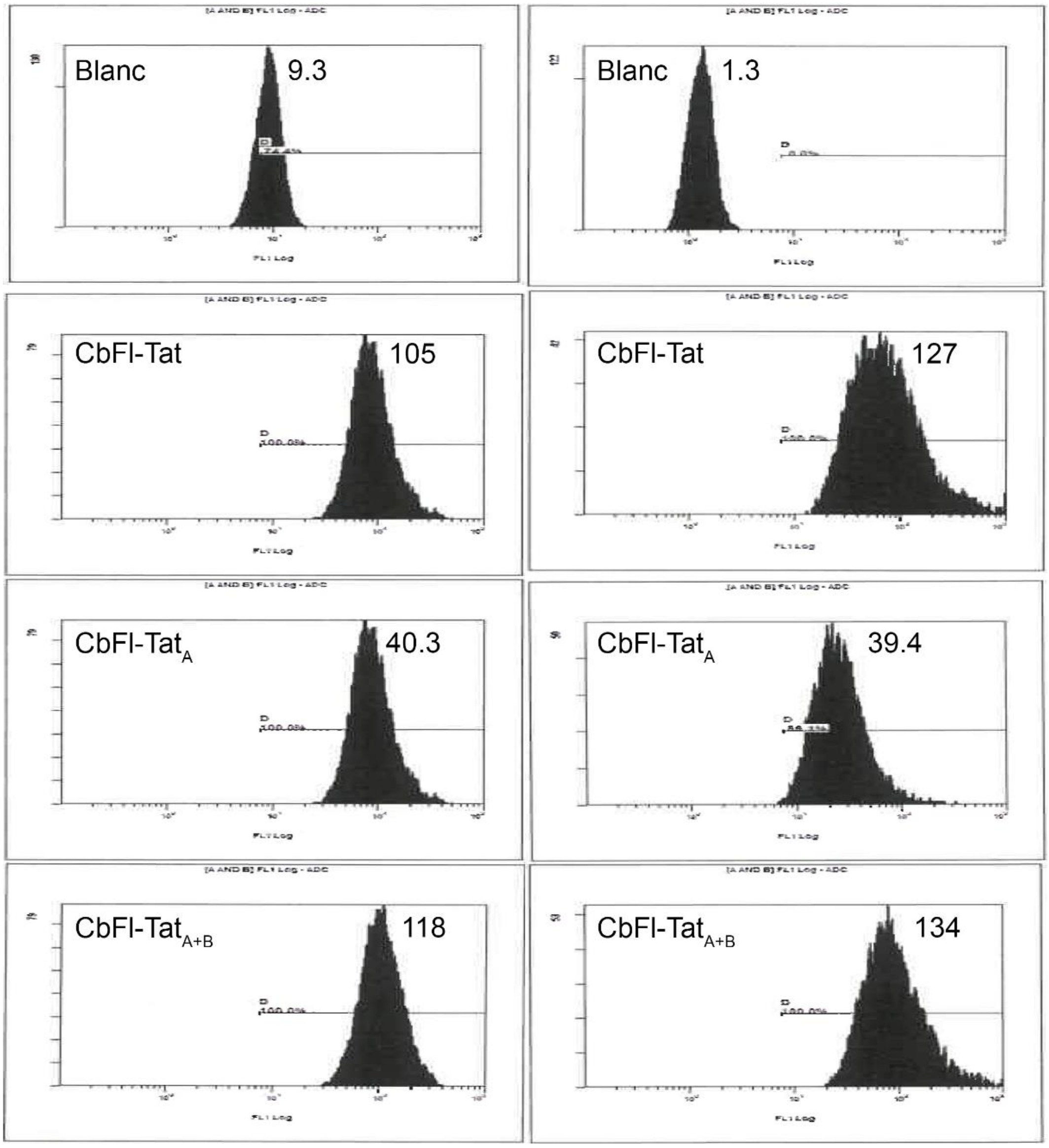

Fig. S1. Representative FACS histograms of fluoresent intensities of Tat peptides at 5 µM (left) and 20 µM (right). Mean intensities are given in the histograms.

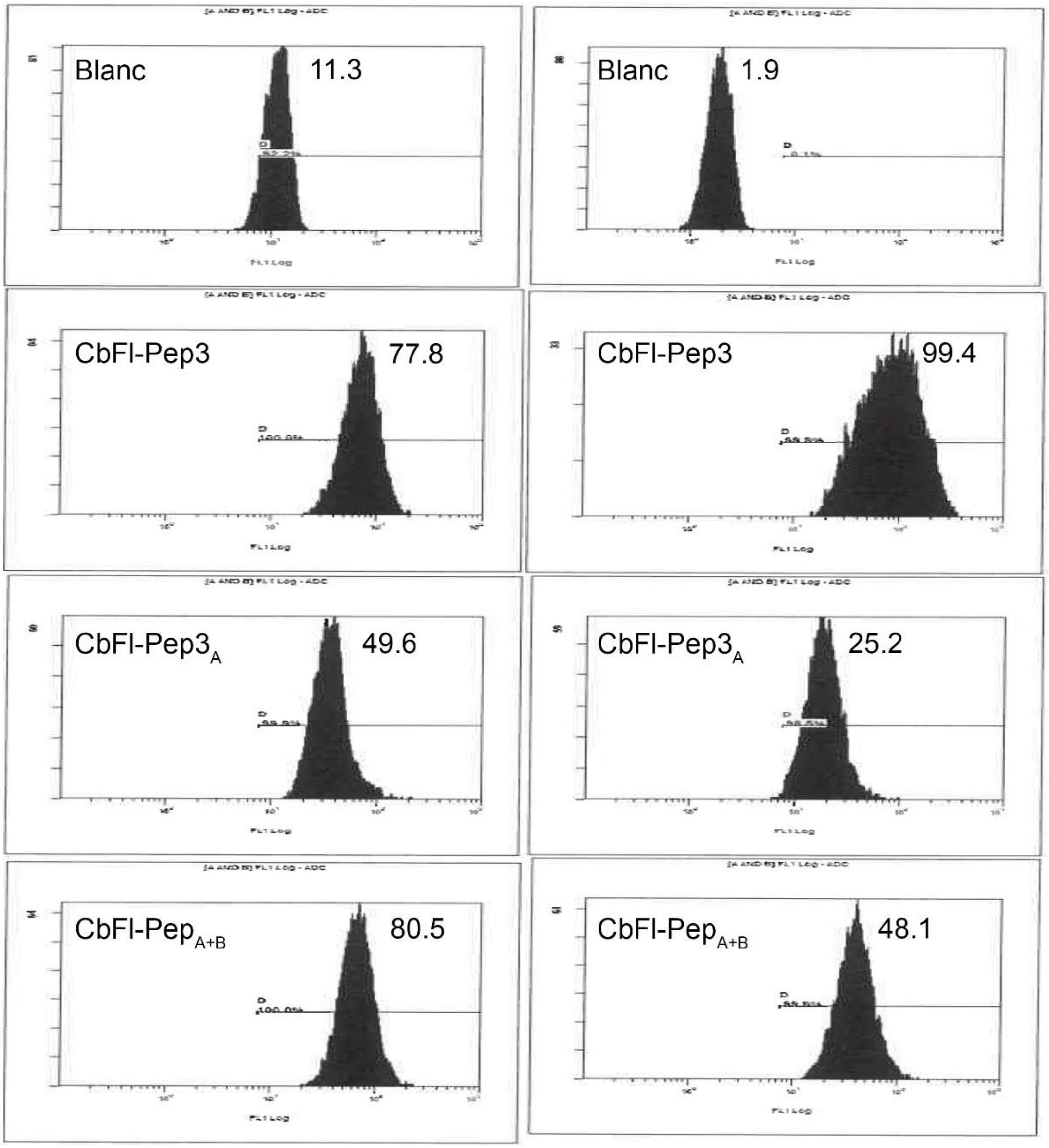

Fig. S2. Representative FACS histograms of fluoresent intensities of Pep-2 peptides at 5 µM (left) and 20 µM (right). Mean intensities are given in the histograms.

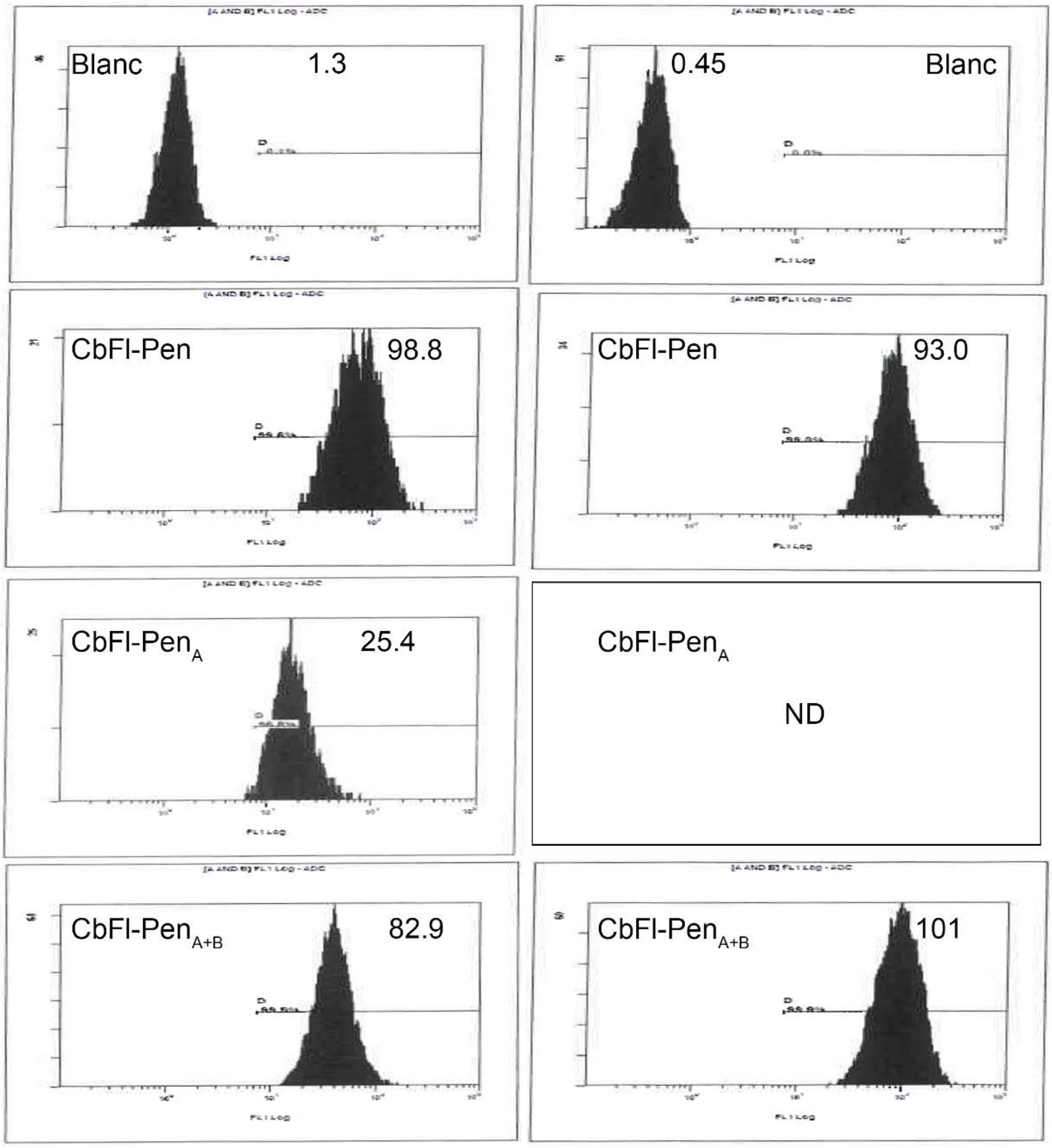

Fig. S3. Representative FACS histograms of fluoresent intensities of Penetratin peptides at 5 μM (left) and 20 μM (right). Mean intensities are given in the histograms.

Viability studies on the peptides

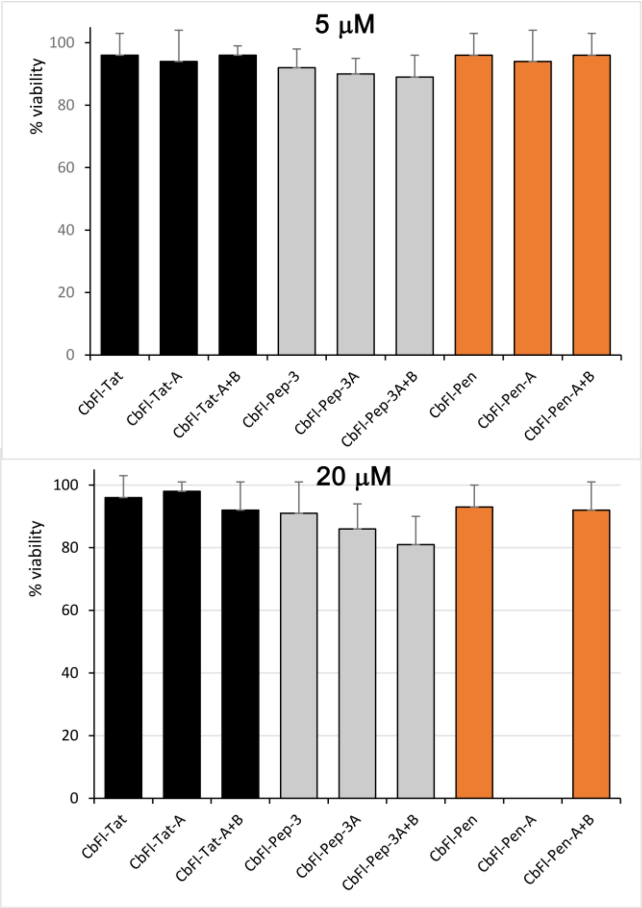

Fig. S4. Results of the toxicity (WST-8) tests at 5 µM (top) and 20 µM (bottom).

Structures

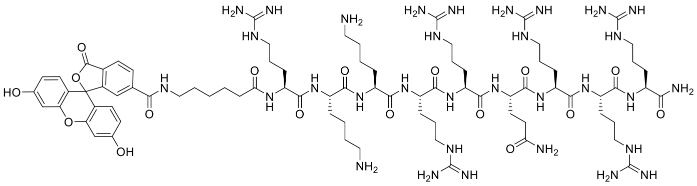

CbFl-Tat

Chemical Formula: $C_{80}H_{128}N_{32}O_{17}$
Exact Mass: 1809.01
Molecular Weight: 1810.11

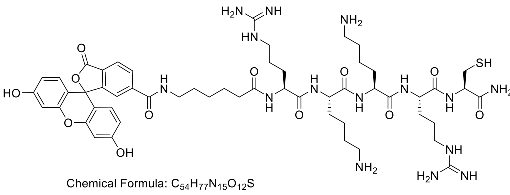

Chemical Formula: $C_{54}H_{77}N_{15}O_{12}S$
Exact Mass: 1159.56
Molecular Weight: 1160.36

CbFl-Tat$_A$

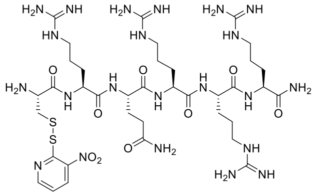

Chemical Formula: $C_{37}H_{66}N_{22}O_9S_2$
Exact Mass: 1026.48
Molecular Weight: 1027.20

c-Tat$_B$

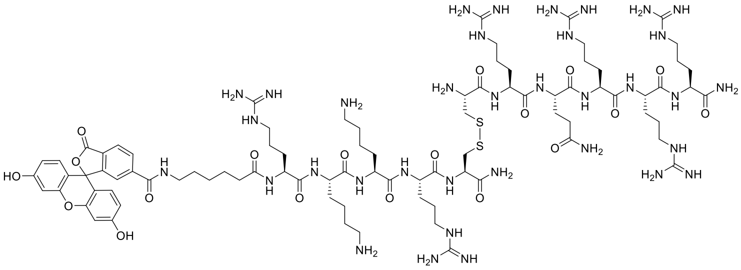

Chemical Formula: $C_{86}H_{139}N_{35}O_{19}S_2$
Exact Mass: 2030.04
Molecular Weight: 2031.40

CbFl-Tat$_{A+B}$

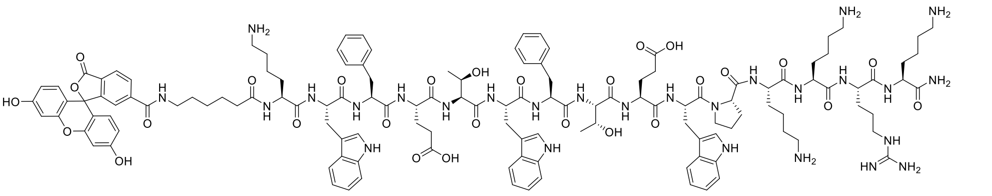

Chemical Formula: $C_{131}H_{167}N_{27}O_{28}$
Exact Mass: 2566.25
Molecular Weight: 2567.94

CbFl-Pep-3

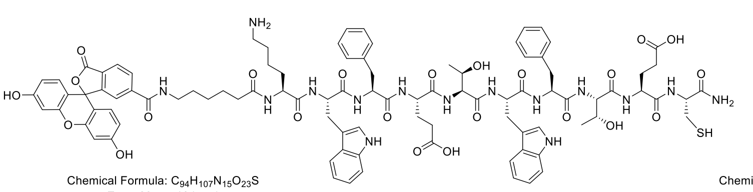

Chemical Formula: $C_{94}H_{107}N_{15}O_{23}S$
Exact Mass: 1845.74
Molecular Weight: 1847.03

CbFl-Pep-3$_A$

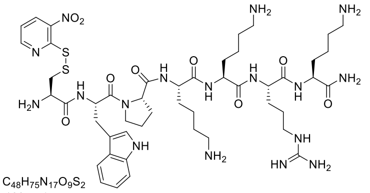

Chemical Formula: $C_{48}H_{75}N_{17}O_9S_2$
Exact Mass: 1097.54
Molecular Weight: 1098.36

c-Pep-3$_B$

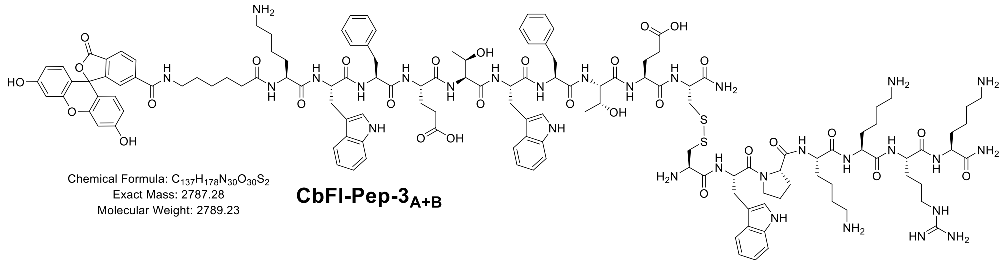

Chemical Formula: $C_{137}H_{178}N_{30}O_{30}S_2$
Exact Mass: 2787.28
Molecular Weight: 2789.23

CbFl-Pep-3$_{A+B}$

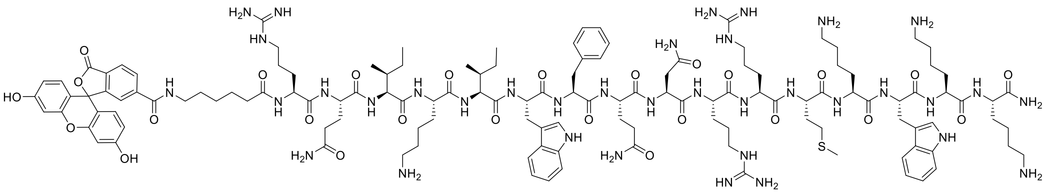

Chemical Formula: $C_{131}H_{190}N_{36}O_{26}S$
Exact Mass: 2715.44
Molecular Weight: 2717.25

CbFl-Pen

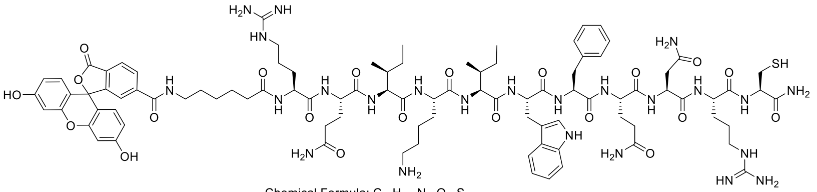

Chemical Formula: $C_{94}H_{128}N_{24}O_{21}S$
Exact Mass: 1960.94
Molecular Weight: 1962.27

CbFl-Pen$_A$

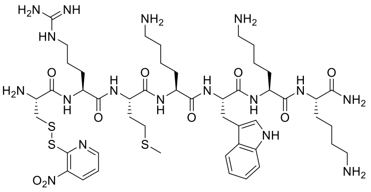

Chemical Formula: $C_{48}H_{77}N_{17}O_9S_3$
Exact Mass: 1131.53
Molecular Weight: 1132.43

c-Pen$_B$

CbFl-Pen$_{A+B}$

Chemical Formula: $C_{137}H_{201}N_{39}O_{28}S_3$
Exact Mass: 2936.47
Molecular Weight: 2938.54

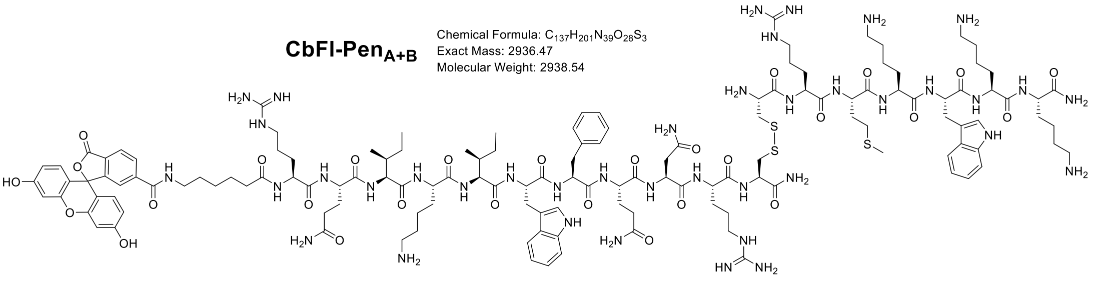

Fig. S5. Structures of all compounds used in this study.

Analyses

HPLC chromatograms and mass spectra

CbFl-Tat

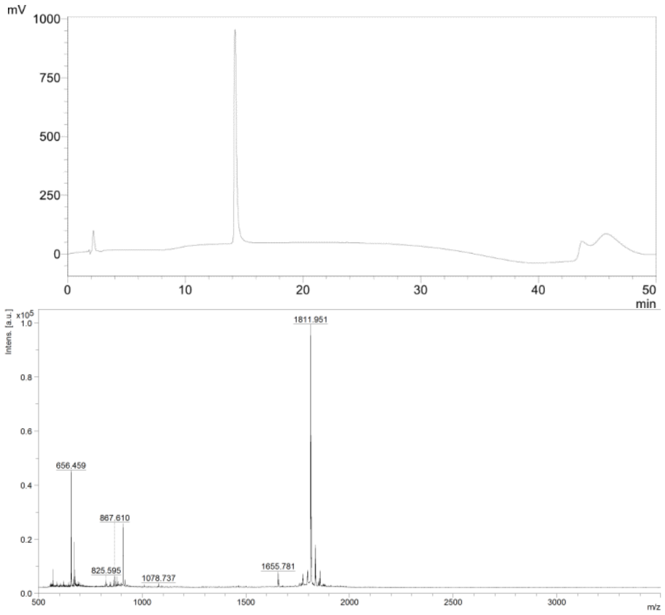

CbFl-Tat$_A$

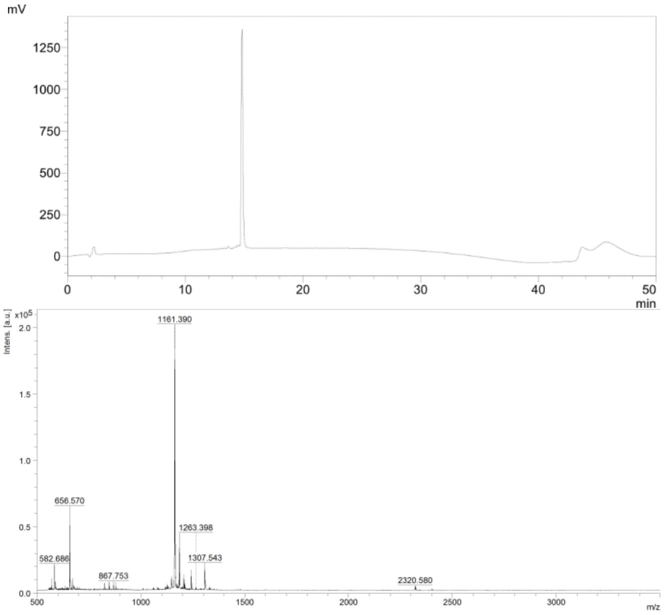

c-Tat$_B$

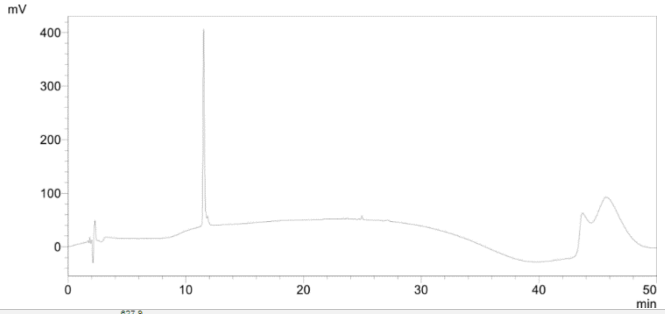

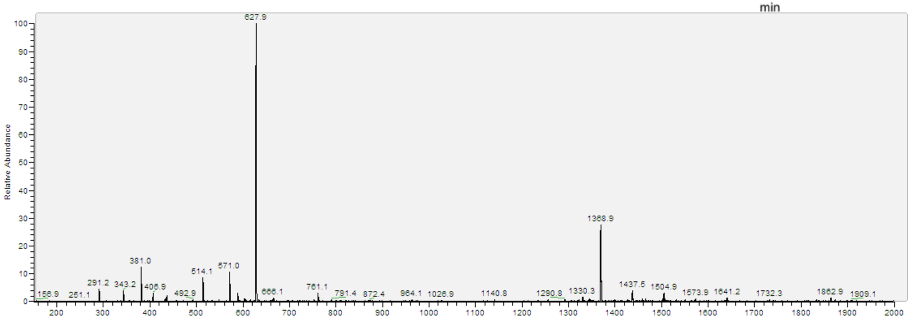

CbFl-Tat$_{A+B}$

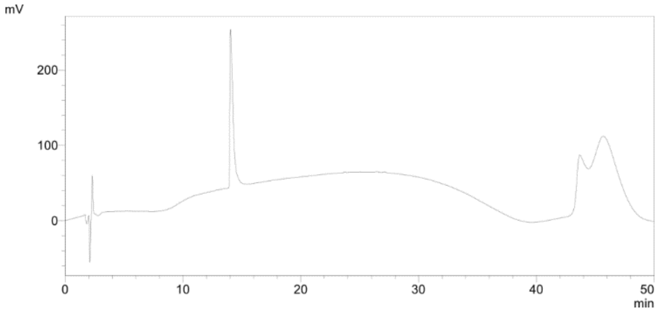

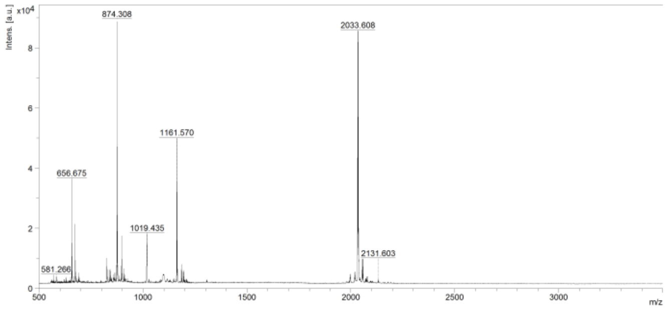

CbFl-Pep-3

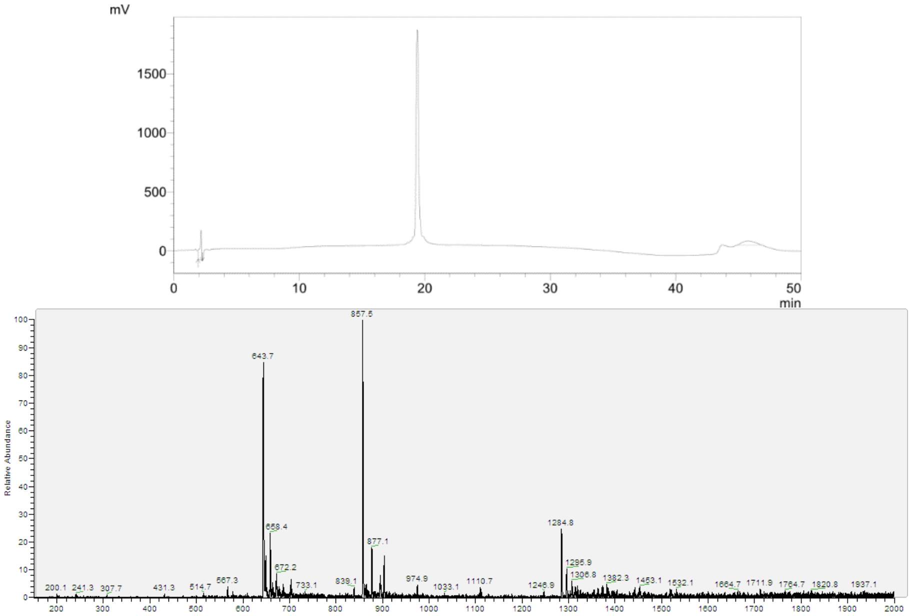

CbFl-Pep-3_A

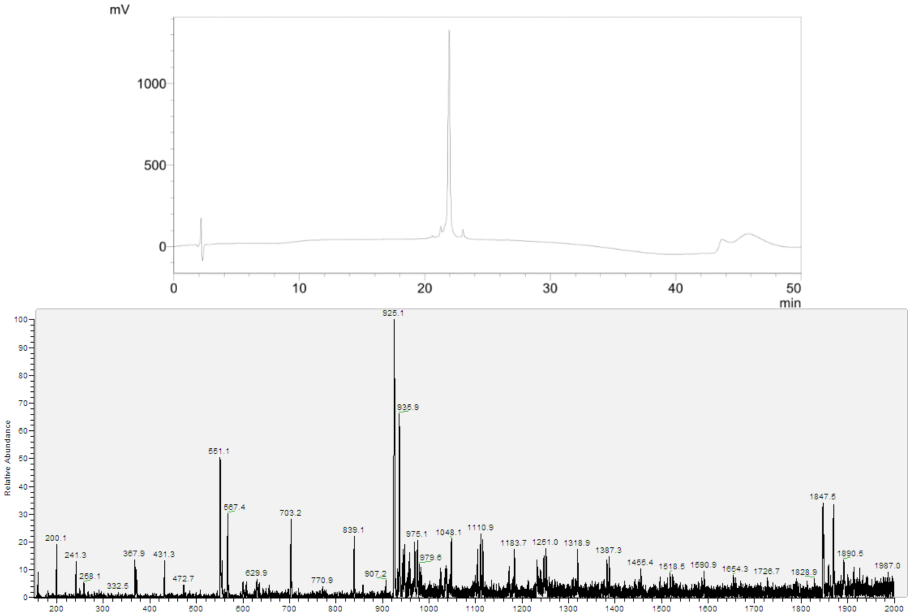

c-Pep-3$_B$

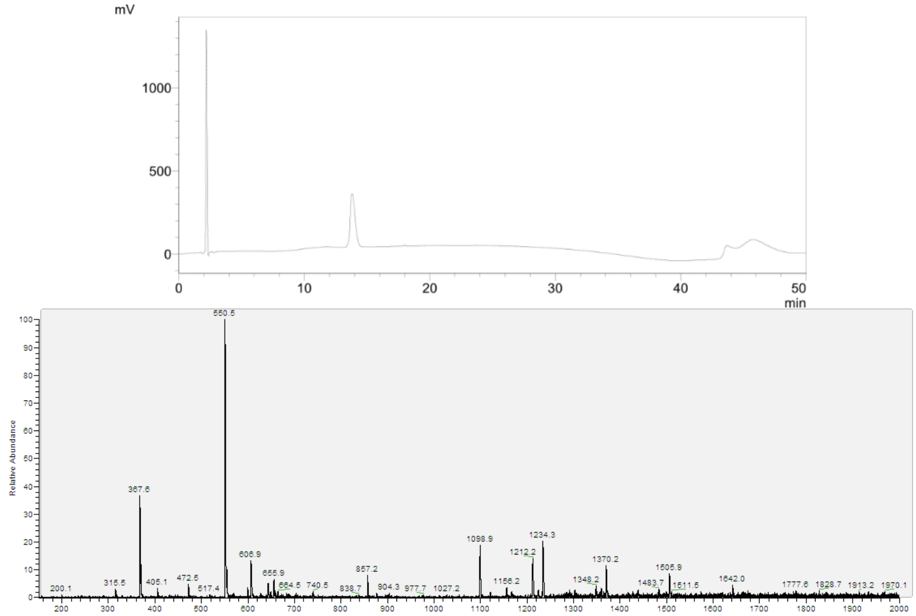

CbFl-Pep3$_{A+B}$

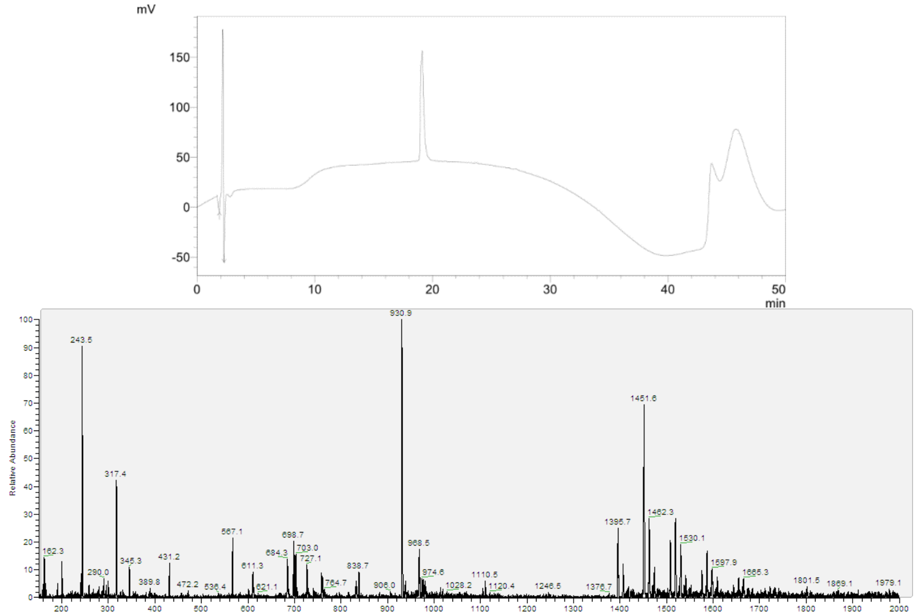

CbFl-Pen-3

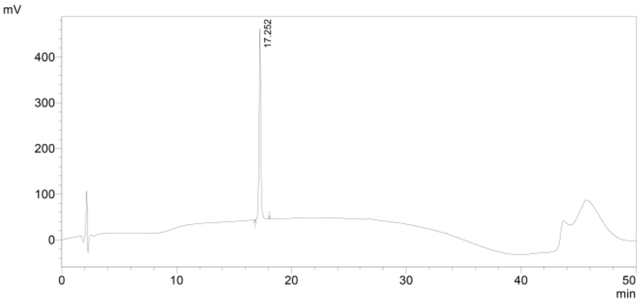

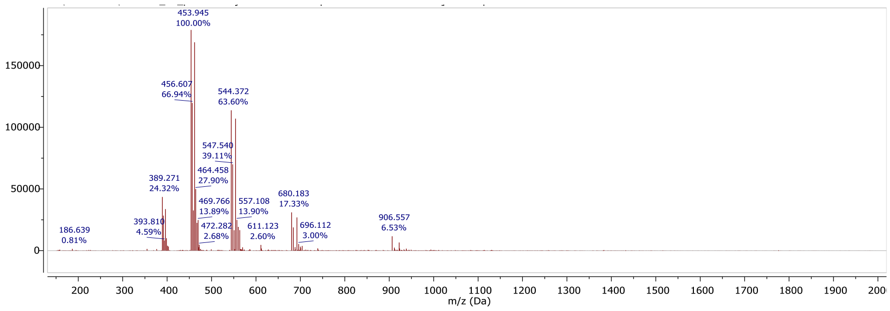

CbFl-Pen-3_A

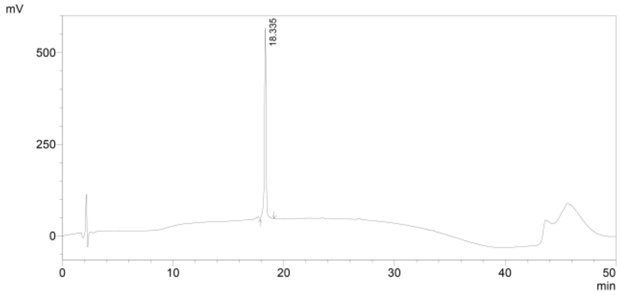

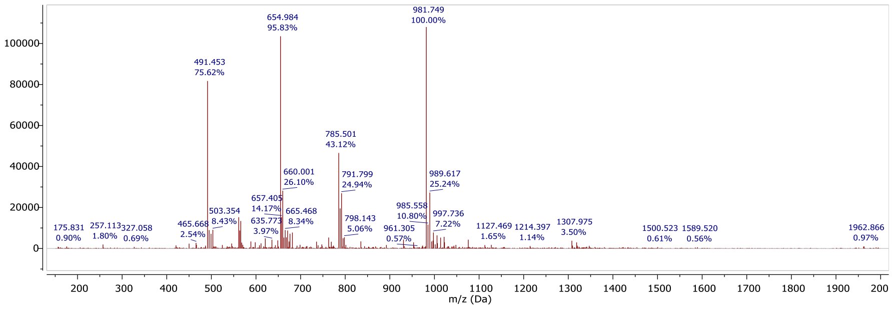

c-Pen-3_B

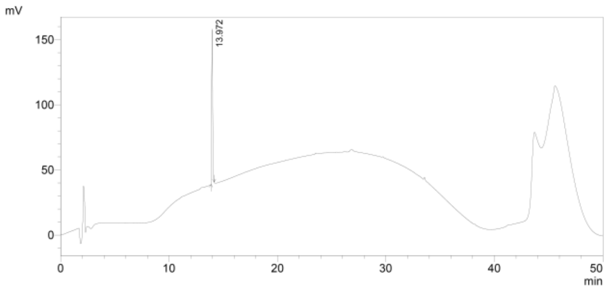

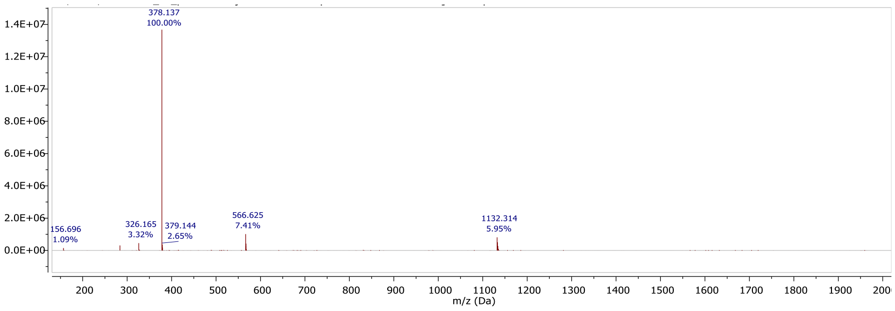

CbFl-Pen-3_{A+B}

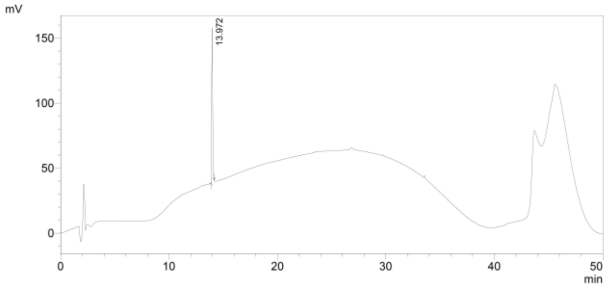

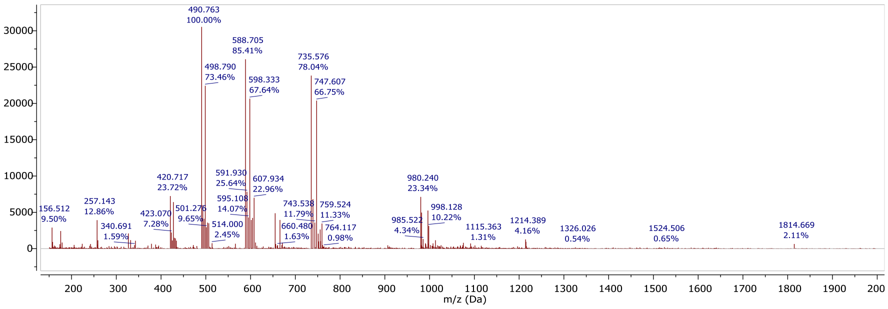

Supplement: Supplementary file 1 — Additional preparation procedures and characterization (viability study, HPLC, Mass analysis) of the peptides, can be found in the electronic supplementary information (PDF 1664 kb) [file 726_2020_2880_MOESM1_ESM.pdf]
